# Pressure overload by suprarenal aortic constriction in mice leads to left ventricular hypertrophy without c-Kit expression in cardiomyocytes

**DOI:** 10.1038/s41598-020-72273-3

**Published:** 2020-09-18

**Authors:** Amy M. Nicks, Scott H. Kesteven, Ming Li, Jianxin Wu, Andrea Y. Chan, Nawazish Naqvi, Ahsan Husain, Michael P. Feneley, Nicola J. Smith, Siiri E. Iismaa, Robert M. Graham

**Affiliations:** 1 Division of Molecular Cardiology and Biophysics, Victor Chang Cardiac Research Institute, 405 Liverpool Street, Darlinghurst, Sydney, NSW 2010 Australia; 2 St Vincent’s Clinical School, University of New South Wales, Sydney, NSW 2052 Australia; 3 Department of Medicine, Emory University, Atlanta, GA 30322 USA; 4 Present Address: Cardiac Regeneration Research Institute, Wenzhou Medical University, Wenzhou, 325035 China

**Keywords:** Cardiovascular biology, Cardiac hypertrophy, Cardiac regeneration, Cardiovascular diseases, Physiology, Cardiovascular biology, Cardiac hypertrophy, Cardiac regeneration, Cardiovascular diseases

## Abstract

Animal models of pressure overload are valuable for understanding hypertensive heart disease. We characterised a surgical model of pressure overload-induced hypertrophy in C57BL/6J mice produced by suprarenal aortic constriction (SAC). Compared to sham controls, at one week post-SAC systolic blood pressure was significantly elevated and left ventricular (LV) hypertrophy was evident by a 50% increase in the LV weight-to-tibia length ratio due to cardiomyocyte hypertrophy. As a result, LV end-diastolic wall thickness-to-chamber radius (h/R) ratio increased, consistent with the development of concentric hypertrophy. LV wall thickening was not sufficient to normalise LV wall stress, which also increased, resulting in LV systolic dysfunction with reductions in ejection fraction and fractional shortening, but no evidence of heart failure. Pathological LV remodelling was evident by the re-expression of fetal genes and coronary artery perivascular fibrosis, with ischaemia indicated by enhanced cardiomyocyte *Hif1a* expression. The expression of stem cell factor receptor, c-Kit, was low basally in cardiomyocytes and did not change following the development of robust hypertrophy, suggesting there is no role for cardiomyocyte c-Kit signalling in pathological LV remodelling following pressure overload.

## Introduction

Heart failure is a common end-result of a variety of cardiovascular diseases, including ischaemic and hypertensive heart disease. Hypertension is the primary cause of pathological left ventricular (LV) hypertrophy in 75% of patients^1^. While the hypertrophic response is initially compensatory to maintain heart function in the face of pressure overload (PO), pathological hypertrophy eventually becomes decompensatory, with decreased cardiac performance that precedes overt heart failure. Current treatments for heart failure merely slow the progression to end-stage heart failure, since there is no cure other than heart transplantation. Animal models of hypertensive heart disease are, thus, critical to developing potential new regenerative therapies^2^.

Two commonly used cardiac injury models are myocardial infarction (MI) produced by ligation of the left anterior descending coronary artery, and aortic constriction; the latter first developed and characterised by Rytand^3^ and Goldblatt et al.^4^, produced by ligating the descending abdominal aorta between or immediately above the renal arteries. Constriction of the abdominal aorta induces PO leading to the rapid development of cardiac hypertrophy and heart failure^2,5^. Historically, aortic constriction models were developed in larger animal species, including rabbits, dogs, guinea pigs and rats^2,5^, but over the last few decades these techniques have been adapted to mice- the preferred species for genetic studies that are also economical^6,7^. Thoracic aortic constriction (TAC) is the most common PO model in mice that involves ligation of the aorta at the level of the arch and requires microsurgical expertise (intubation, a thoracotomy and retraction of the chest wall). The severity of PO models vary depending on the degree (needle size) of constriction and tightness of suture tie, the location and duration of banding, and strain and sex of animal^8^. Currently, the standard model of PO in rats is aortic constriction of the abdominal aorta, and despite being less technically demanding than TAC, is not well studied in mice.

c-Kit is a type III transmembrane tyrosine kinase receptor. Upon binding to stem cell factor (SCF) it activates a downstream signalling cascade required for mast cell and melanocyte development, haematopoiesis, and differentiation of spermatogonial stem cells. c-Kit expression has been evaluated in many endogenous cardiac cell populations, such as mast cells^9^, cardiac stem cells^10^, endothelial and endothelial progenitor cells^9,11^, and cardiomyocytes^12^. Studies using c-Kit knock-in mice for genetic-linage tracing have evaluated c-kit as a stem cell marker and reported a very low expression of c-Kit in cardiomyocytes at baseline and following MI^11–14^. However, c-Kit expression determined using a transgenic c-Kit reporter system identified its expression in a subpopulation of extant cardiomyocytes that increased after isoproterenol-induced cardiac injury^15^. The controversies surrounding cardiac c-Kit expression are complicated further by the use of many different genetically modified c-Kit mouse models, cardiac injury types, and methods used to identify cardiac cell populations, which have been extensively reviewed^16,17^.

We have previously reported that c-Kit is expressed transiently in cardiomyocytes beginning immediately after birth and terminating a few days later, coincident with exit of cardiomyocytes from the cell cycle^18^. Moreover, when subjected to PO, Kit^W/Wv^ mice (which have global inhibition of c-Kit signalling from conception) had enhanced cardiac contractile function that was directly related to the degree of cardiomyocyte proliferation, and improved survival. Taken together, these findings suggested that c-Kit signalling might function to actively block cardiomyocyte proliferation. We, thus, questioned if the failure to initiate cardiomyocyte proliferation in wild-type mice, in response to pressure overload, is due to reactivation of c-Kit; cardiomyocyte c-Kit re-expression having been reported in response to another stressor, myocardial cryoablation injury^19^. To this end we characterised a model of PO due to suprarenal aortic constriction (SAC), which induces hypertension and is a clinically relevant model of hypertensive heart disease^20^, and used it to evaluate cardiomyocyte c-Kit expression in wild-type mice. We show that despite SAC resulting in the rapid onset of PO-induced hypertension, which is associated with pathological cardiac hypertrophy, significant LV wall stress, perivascular fibrosis and ischaemia, cardiomyocyte c-Kit expression is barely detectable before, and is unchanged after the development of PO.

## Results

### SAC causes increased LV wall stress and systolic dysfunction

SAC surgery was considered a priori to be successful if it resulted in peak systolic aortic pressure (SAP) more than two standard deviations above that of sham-operated mice (109 mmHg ± 10 mmHg), i.e. SAP > 129 mmHg, and did not result in signs of heart failure (Supplementary Fig. S2). Our success rate for the induction of hypertension with SAC was 82% (36/44), which is greater than that reported in TAC studies (70%)^21^ or SAC (67%) in rats^22^. Thus, a total of eight mice were excluded from this study: four were part of a cohort in which LV tissue was harvested and four were part of a cohort in which cardiomyocytes were isolated. The correlation between SAP and LV weight-to-tibia length (LVW/TL) confirmed the SAC mice included in this study had significant LV hypertrophy and elevated SAP with our exclusion criteria applied (R^2^ = 0.92; Supplementary Fig. S2).

**Figure 1:**
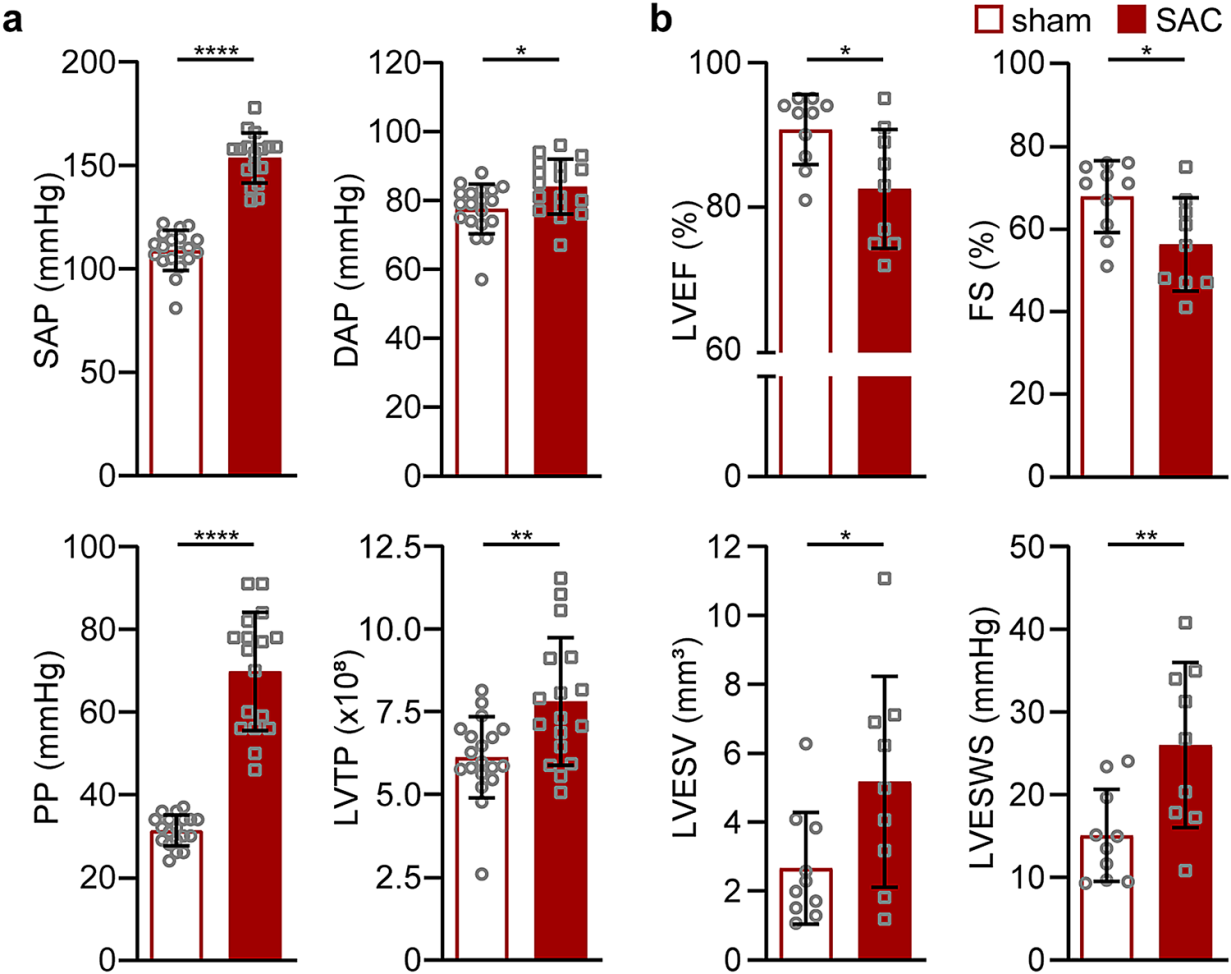
Hypertension phenotype after SAC-induced pressure overload. (**a**) Hemodynamic measurements were recorded one week post-surgery (sham, n = 18; SAC, n = 15) in adult male C57BL/6J mice (9-week-old) and analyzed using AcqKnowledge software (v3.8). *SAP* systolic aortic pressure, *DAP* diastolic aortic pressure, *PP* pulse pressure, *LVTP* LV triple product (mmHg^2^/s^2^), *LVEF* LV ejection fraction. (**b**) Echocardiography was performed six days post-sham or -SAC surgery (sham, n = 11; SAC, n = 9) in adult male C57BL/6J mice (9-week-old). *EF* ejection fraction, *FS* fractional shortening, *LVESV* LV end-systolic volume, *LVESWS* LV end-systolic wall stress. Data are presented as means ± SD; independent comparisons were made by two-tailed Student’s unpaired t-tests; *P < 0.05, **P < 0.01, and ****P < 0.0001.

SAC caused peak SAP to rise by an average of + 45 mmHg after one week (P < 0.0001; Fig. 1a). End-diastolic and mean aortic pressure rose (+ 6 mmHg, P = 0.015, and + 20 mmHg, P < 0.0001, respectively; Table 1). Consistent with these changes, pulse pressure increased 126% (P < 0.0001; Fig. 1a) post-SAC. These aortic pressure changes were associated with increases in peak LV systolic pressure post-SAC (+ 38 mmHg LVSP, P < 0.0001; Table 1). SAC also resulted in increases in LV triple product^23^, an index of myocardial oxygen consumption and in LV cardiac workload (+ 28%, P = 0.003; Fig. 1a), however, cardiac contractility (dP/dt_max_) and relaxation (dP/dt_min_) were similar between sham and SAC groups (Table 1), as was heart rate, indicating that the increased LVSP was the main determining factor for the increase in LV triple product. SAC also induced small decreases in both LV ejection fraction (− 8%, P = 0.033; Fig. 1b) and fractional shortening (− 11%, P = 0.022; Fig. 1b), although cardiac output was unaffected, and there was no evidence of congestive heart failure as lung weights were comparable between sham and SAC groups (Table 1). LV end-diastolic volume (LVEDV) was unchanged post-SAC, with a small increase in LVEDP that remained within the normal range (9.6 ± 3 mmHg, P = 0.039; Table 1). However, end-systolic wall stress increased by + 73% (P < 0.0001; Fig. 1b) one week after SAC, associated with a significant increase in LV end-systolic volume (LVESV, P = 0.037; Fig. 1b). Together, these findings show that SAC induces hypertension with increases in LV workload and wall stress, resulting in impaired systolic function but largely preserved diastolic function, and no evidence of heart failure.

**Table 1:** Gross morphology, micromanometry and echocardiography post-SAC.

	Sham	SAC	p-value	
**Gross morphology**	n = 7	n = 6		
BW (g)	22.29 ± 1.1	21.92 ± 1.17	0.566	ns
HW (mg)	117 ± 11.37	161.5 ± 10.85	P < 0.0001	****
LV weight (mg)	77.66 ± 7.06	111.4 ± 6.68	P < 0.0001	****
TL (mm)	16.03 ± 0.34	15.61 ± 0.59	0.131	ns
Lungs (mg)	135.2 ± 10.7	126.5 ± 8.3	0.134	ns
HW/BW (mg/g)	5.26 ± 0.51	7.39 ± 0.65	P < 0.0001	****
Lungs/TL (mg/mm)	8.44 ± 0.69	8.57 ± 1.35	0.82	ns
**Micromanometry**	n = 19	n = 17		
HR (bpm)	494 ± 15	490 ± 17	0.282	ns
MAP (mmHg)	88 ± 8	108 ± 6	P < 0.0001	****
LV dP/dt_max_ (mmHg/S)	11,491 ± 1746	11,048 ± 1912	0.473	ns
LV dP/dt_min_ (mmHg/S)	− 8,044 ± 1,268	− 7,695 ± 1589	0.469	ns
LVSP (mmHg)	107 ± 8	145 ± 12	P < 0.0001	****
LVEDP (mmHg)	7.7 ± 2.45	9.6 ± 3	0.039	*
LV HR (bpm)	492 ± 12	481 ± 21	0.447	ns
**Echocardiography**	n = 10	n = 9		
HR (bpm)	501 ± 48	478 ± 42	0.3	ns
LVEDV (mm^3^)	27.6 ± 2.9	28.3 ± 5.7	0.745	ns
SV (µl)	24.9 ± 2.1	23.1 ± 3.5	0.186	ns
CO (ml/min)	12.5 ± 1.7	11 ± 1.4	0.054	ns
h_end-diastole_ (mm)	1.026 ± 0.069	1.221 ± 0.092	P < 0.0001	****
R_end-diastole_ (mm)	1.54 ± 0.076	1.54 ± 0.143	0.935	ns
h_end-systole_ (mm)	1.614 ± 0.256	1.775 ± 0.186	0.14	ns
R_end-systole_ (mm)	0.498 ± 0.151	0.681 ± 0.225	0.068	ns
h/R_end-systole_ (mm)	3.707 ± 1.224	2.987 ± 1.445	0.256	ns
ESP (2 × SAP + DAP)/3	100 ± 5	132 ± 9	P < 0.0001	****
LVEDWS (LVEDP × R_end-diastole_ / 2 × h_end-diastole_)	6.05 ± 2.34	6.41 ± 1.93	0.78	ns

Gross morphology, micromanometry, and echocardiography from adult male C57BL/6J mice (9-week-old) at one week post-sham or -SAC surgery. Data are presented as mean ± SD; independent comparisons were made by two-tailed Student’s unpaired t-tests; *P < 0.05, **P < 0.01, and ****P < 0.0001 and ns, non-significant.

*BW* body weight, *HW* heart weight, *TL* tibia length, *HR* heart rate, *MAP* mean aortic pressure, *dP/dt*_*max*_ maximal rate of contractility, *dP/dt*_*min*_ minimal rate of contractility, *LVSP* LV systolic pressure, *LVEDP* end-diastolic blood pressure, *LVEDV* LV end-diastolic volume, *SV* stroke volume, *CO* cardiac output, *h* LV wall thickness, *R* LV internal chamber radius, *LVESP* LV end-systolic pressure, *LVEDWS* LV end-diastolic wall stress.

### Pressure overload results in concentric LV hypertrophy

**Figure 2:**
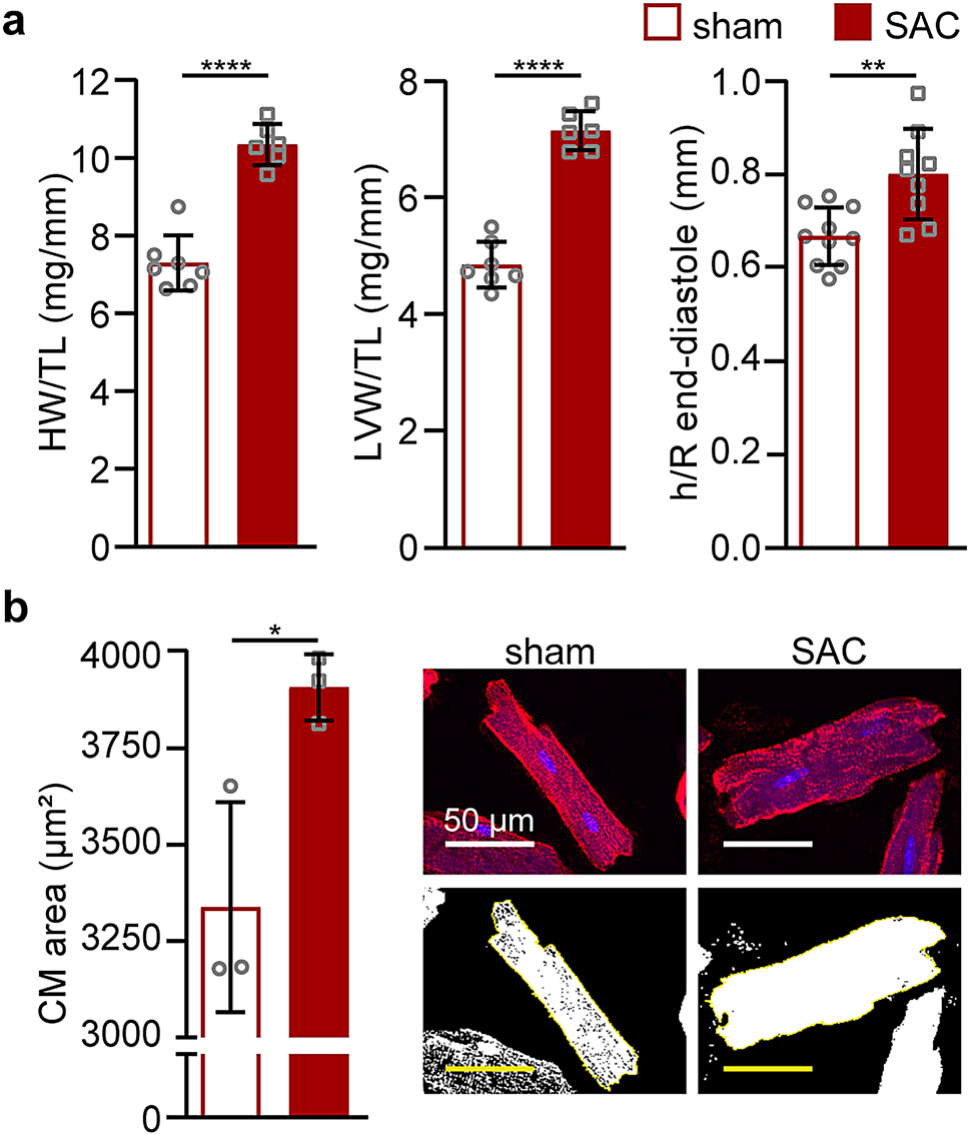
SAC-induced pressure overload leads to LV and cellular hypertrophy. Gross morphology measurements were made at one week after sham or SAC surgery in adult male C57BL/6J mice (9-week-old). (**a**) Heart and LV weights were normalized to TL (sham, n = 7; SAC, n = 6). LV wall thickness (h) to internal LV chamber radius (R) ratio dimensions were measured by echocardiography six days post-surgery (sham, n = 10; SAC, n = 9). (**b**) Cardiomyocyte (CM) areas were measured using ImageJ (n = 3 mice per group, 30–40 binucleated CM areas were measured) and representative images are shown. Top, CM membranes were stained with laminin (red) and DNA with DAPI (blue); bottom, replica binary images show the cell area (white) within the outline of each CM measured using ImageJ. Data are presented as means ± SD; independent comparisons were performed using two-tailed Student’s unpaired *t*-tests; *P < 0.05, **P < 0.01, and ****P < 0.0001. *LV* left ventricle, *HW* heart weight, *TL* tibia length.

Heart weight-to-tibia length (HW/TL) and LVW/TL ratios were 42% and 48% higher after SAC, respectively, compared to those in the sham animals (P < 0.0001; Fig. 2a). Heart growth involved a 20% increase in the ratio of the LV wall thickness-to-chamber radius (h/R) at end-diastole, consistent with the development of concentric hypertrophy post-SAC (P = 0.002; Fig. 2a). Hypertrophy was also evident at the cellular level as cardiomyocyte area increased by 17% in SAC relative to sham animals (Fig. 2b). Hence, SAC-induced hypertension leads to robust hypertrophy in this model of hypertensive heart disease.

### Pathological LV remodelling is evident post-SAC

**Figure 3:**
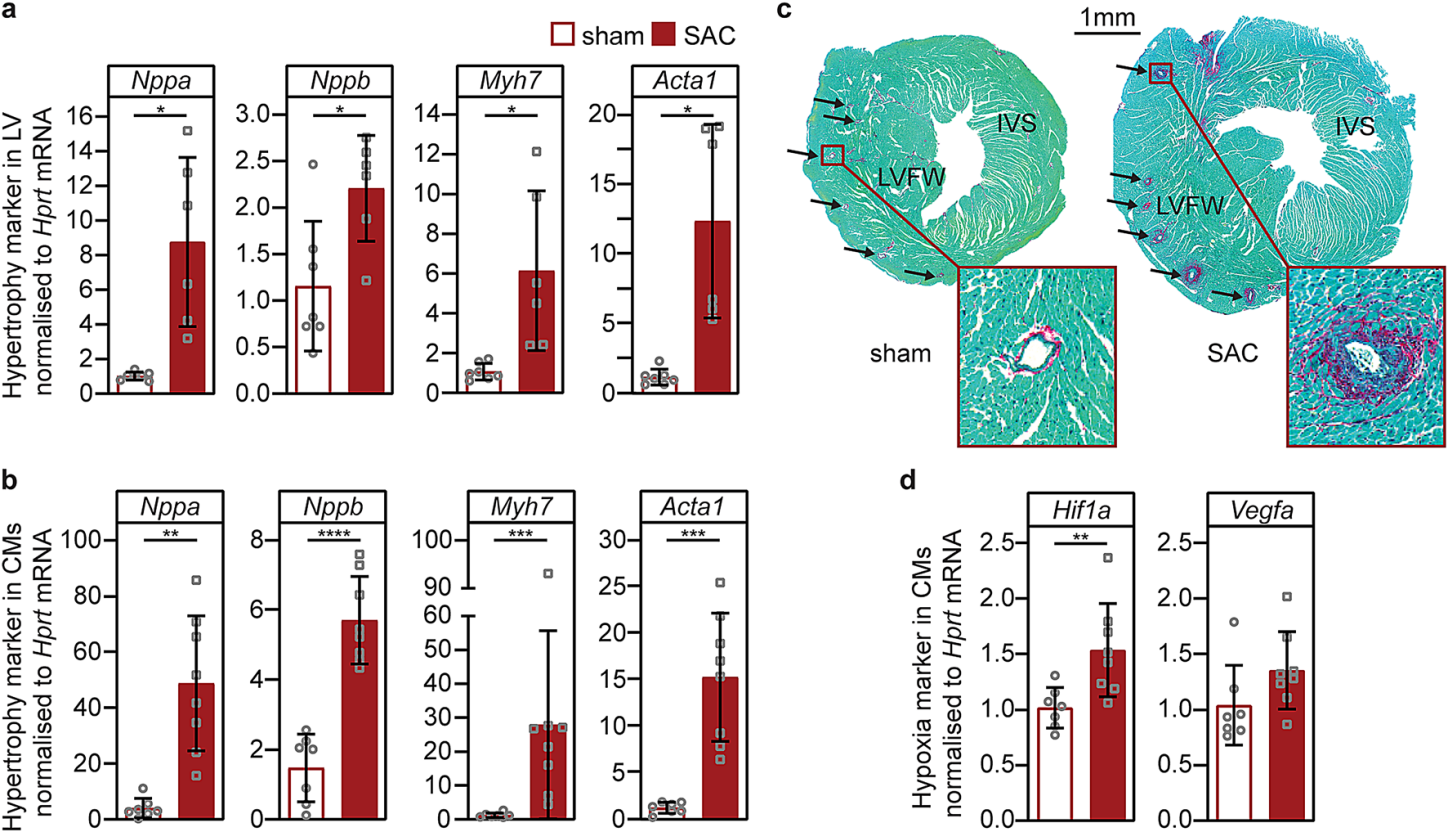
Pathological LV hypertrophy and perivascular fibrosis post-SAC. Pathological hypertrophy marker mRNAs were evaluated one week post-sham or -SAC surgery in adult male C57BL/6J mice (9-week-old) from (**a**) LV tissue (sham, n = 7; SAC, n = 6) or (**b**) enriched cardiomyocyte fractions (sham, n = 7; SAC, n = 8). (**c**) Representative transverse LV sections demonstrate the development of perivascular fibrosis around the coronaries (indicated by black arrows) after SAC-surgery (right) compared to sham-surgery (left). Sections were stained with Fast Green and Picrosirius Red to identify the cytoplasm and collagen, respectively. (**d**) *Hif1a* and *Vegfa* expression in cardiomyocytes one week post-SAC (sham, n = 7; SAC, n = 8). Data are presented as means ± SD, independent comparisons were made by two-tailed Student’s unpaired t-tests; *P < 0.05, **P < 0.01, ***P < 0.001 and ****P < 0.0001.

Consistent with the development of pathological hypertrophy in response to PO, expression of fetal genes (*Nppa,* ANP; *Nppb*, BNP; *Myh7*, β-MHC; and *Acta1*,α-SKA) was significantly increased at one week post-SAC by + 8.6- (P = 0.012), + 1.9- (P = 0.013), + 5.8- (P = 0.027), and + 10.8-fold (P = 0.011) in LV tissues, respectively, and by + 12.4- (P = 0.001), + 3.9- (P < 0.0001), + 24.6- (P = 0.0003) and + 12.6- fold (P = 0.0007) in enriched ventricular cardiomyocytes compared to sham controls (Fig. 3a,b). SAC was also associated with the accumulation of fibrillar collagens type 1 and 3, predominantly around coronary arteries in the LV free wall, rather than interstitial fibrosis, indicating the presence of coronary artery perivascular fibrosis at one week post-SAC (Fig. 3c). Moreover, there was evidence of an ischaemic response to PO as evidenced by enhanced mRNA levels of the transcription factor HIF-1α in cardiomyocytes post-SAC, although at one week, mRNA levels of its downstream target, VEGF-A, had not yet increased (Fig. 3d).

### Renin mRNA increases after one week of SAC

**Figure 4:**
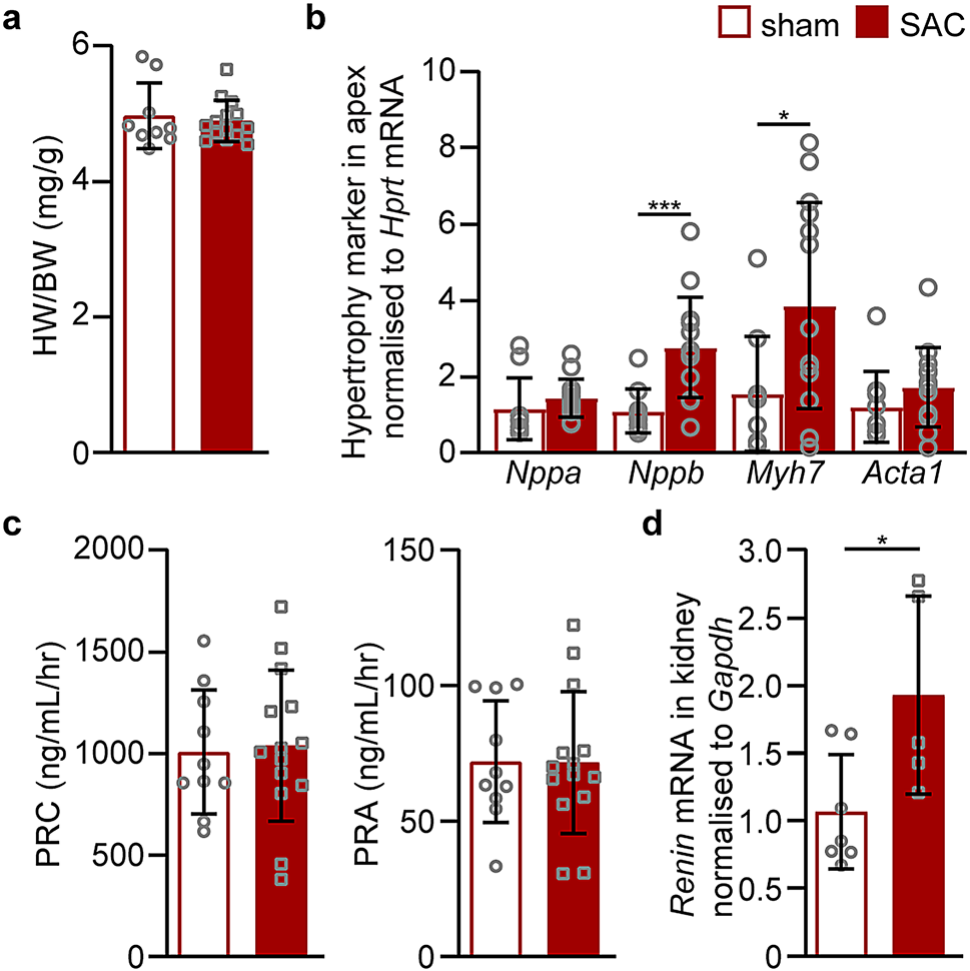
Pathological cardiac remodelling at 24 h post-SAC. Sham and SAC surgeries were performed on adult male C57BL/6J mice at 8–10 weeks of age and tissues were collected at 24 h after surgery. Heart-to-body weight (HW/BW) ratios (**a**) and the expression of pathological hypertrophy markers in the apex of the heart (**b**) were determined at 24 h after sham (n = 10) or SAC (n = 14) surgery. (**c**) Plasma renin concentration (PRC) and activity (PRA) were measured from blood plasma collected at 24 h post-surgery (sham = 10, SAC = 14). (**d**) At one week post-surgery, the expression of renin mRNA was determined in kidney tissues harvested from adult male C57BL/6J mice (9-week-old; sham, n = 7; SAC, n = 5). Data are presented as means ± SD, independent comparisons were made by two-tailed Student’s unpaired t-tests; *P < 0.05, **P < 0.01, ***P < 0.001 and ****P < 0.0001.

The concentration and activity of renin, the rate limiting enzyme of the renin-angiotensin system (RAS), was investigated at 24 h post-SAC. At 24 h, there were no differences in heart-to-body weight ratios (Fig. 4a) and plasma renin concentration (PRC) and activity (PRA) were not different between sham and SAC groups (Fig. 4c), despite the evidence of pathological remodelling indicated by increases in the expression of *Nppb* and *Myh7* in the apex of the heart at this time post-SAC (Fig. 4b). However, at one week post-SAC, *Renin* mRNA was slightly increased in kidney tissues relative to sham controls (Fig. 4d).

### c-Kit is barely detectable in cardiomyocytes and does not change in response to pressure overload

**Figure 5:**
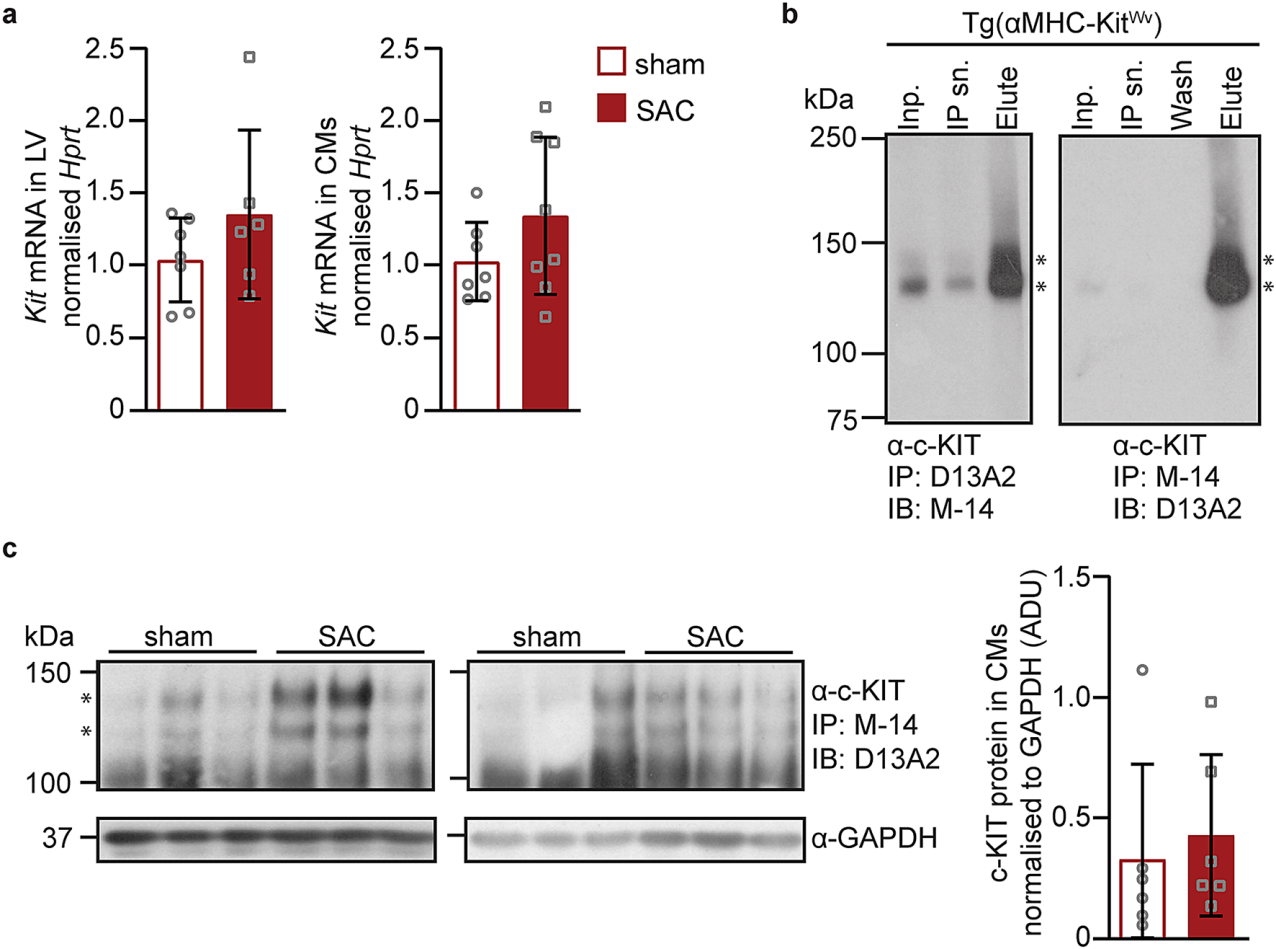
c-Kit mRNA and protein expression following pressure overload. (**a**) At one week post-surgery in adult male C57BL/6J mice (9-week-old) c-Kit mRNA expression (*Kit,* normalized to *Hprt*) in LVs (sham = 7; SAC = 6) or cardiomyocyte-enriched fractions (sham, n = 7; SAC, n = 8) was evaluated following SAC surgery. (**b**) Anti-c-Kit antibody (Ab) specificity was demonstrated by immunoprecipitation (IP) of heart lysates (400 μg) from transgenic mice overexpressing the dominant negative *Wv* c-Kit mutant, Tg(αMHC-Kit^*Wv*^), using either anti-c-Kit D13A2 or M-14 Ab (1:50) followed by size-fractionation and immunoblotting (IB) with the other anti-c-Kit Ab M-14 (1:1,000) or D13A2 (1:500), followed by secondary horseradish peroxidase (HRP) antibodies (1:4,000) and detection using Western Lightning ECL (low HRP sensitivity, Supplementary Fig. S3). Films were exposed to blots for 1 min. Inp., IP input (20 µl); IP sn., IP supernatant (20 µl); IP wash (20 µl); Elute, IP elution from beads (20 µl). (**c**) c-Kit protein was immunoprecipitated from cardiomyocyte lysates (3.5 mg) using anti-c-Kit M-14 (1:50) antibody followed by IB using D13A2 (1:500) anti-c-Kit Ab, and then a secondary HRP antibody (1:10,000). Proteins were detected using Pierce ECL Plus (high HRP sensitivity, Supplementary Fig. S3) at one week post–sham or –SAC surgery (n = 6 per group) from C57BL/6J adult male mice (9-week-old). Films were exposed to blots for 10 min. c-Kit and GAPDH were quantified by densitometry. Full-length gels in Supplementary Fig. S5. Data are presented as means ± SD, independent comparisons were made by two-tailed Student’s unpaired t-tests. No statistically significant differences were observed.

c-Kit mRNA in LV tissues and in enriched ventricular cardiomyocytes was very low (only detectable > 32 cycles of PCR) and did not change post-SAC (Fig. 5a). c-Kit protein was detected in three transgenic mouse lines with cardiomyocyte-specific expression of either wild-type c-Kit [Tg(αMHC-*Kit.1*) and Tg(αMHC-*Kit.2*)] or the dominant-negative c-Kit^*Wv*^ mutant (T660M) [Tg(αMHC-Kit^*Wv*^)]^18,24^, from birth (Supplementary Fig. S3). Expression of the Kit^Wv^ mutation under the control of the α-MHC promoter leads to the predominance of an immature species of c-Kit protein (Supplementary Fig. S3). Immunocytochemistry of isolated Tg(αMHC-Kit^*Wv*^) cardiomyocytes indicated localisation of c-Kit protein in the perinuclear region and not at the cell surface (Supplementary Fig. S4), suggesting protein misfolding. c-Kit could not be detected in wild-type C57BL/6J hearts or cardiomyocyte lysates (100 µg protein) by Western blotting (Supplementary Fig. S3) or in wild-type cardiomyocytes by immunocytochemistry (Supplementary Fig. S4). Immunoprecipitation was then employed to concentrate c-Kit protein from lysates for subsequent detection by Western blotting of SDS-PAGE fractionated immunoprecipitants. Two different commercial anti-c-Kit antibodies were first validated using Tg(αMHC-Kit^*Wv*^) heart lysates (400 µg protein, using a low horseradish peroxidase, HRP, sensitivity ECL kit, Fig. 5b) and c-Kit protein was evident as two bands at 125–145 kDa^25^ (Fig. 5b). Lysates from ventricular cardiomyocytes enriched from entire sham or SAC hearts (3.5 mg protein) were used for immunoprecipitation and only very low levels of c-Kit protein were detected (using a high horseradish peroxidase, HRP, sensitivity ECL kit; Fig. 5c).

## Discussion

Here, we show that SAC-induced PO is a robust model of hypertensive heart disease resulting in elevated blood pressure (154/84 mmHg vs 109/78 mmHg) and a 50% increase in LV weight. This is comparable to the hypertrophy previously reported in other mouse models after two weeks of PO^21,26^ in mice. We demonstrated that LV growth in response to SAC was due to cardiomyocyte hypertrophy, evident by a 17% enlargement in cardiomyocyte area, with an increase in the end-diastolic LV wall thickening-to-chamber radius (h/R ratio); this is consistent with the development of concentric hypertrophy. Despite LV wall thickening, SAC resulted in an increase in LV end-systolic wall stress that was not counterbalanced by hypertrophy, leading to LV systolic dysfunction. This was associated with reductions in LV ejection fraction and fractional shortening without evidence of heart failure. LV remodelling was pathological as evident by the expression of fetal genes and the finding of perivascular fibrosis post-SAC. We found that c-Kit expression was extremely low in adult cardiomyocytes and was not induced in response to SAC.

Very few aortic banding studies report their exclusion criteria^21,22,26–28^, success rate or mortality rate^28^, which precludes proper evaluation of PO models and number of animals required for studies. Our exclusion criterion of > 129 mmHg SAP, representing 2 standard deviations above mean sham SAP, correlated with robust hypertension (a success rate of 82% in SAC-operated mice) and LV hypertrophy producing a consistent pathophysiological phenotype. In our study, induction of SAC was associated with an intra- and post- operative mortality rate of 27%. This is comparable to that reported in rats^22^ and within the range to that reported in TAC models (10–50%)^8,21,28,29^.

A drawback of PO models involving aortic constriction is variability in the degree of stenosis and tightness of tie. This is controlled to some extent by ligating the aorta onto a fixed gauge needle and then removing the needle. Recent developments in the TAC method employ use of an O-ring to control the tightness and degree of ligation without temporary occlusion of the aorta^30^. Another more robust TAC method^28^ assesses the aortic lumen of individual mice by echocardiography to calibrate a custom-sized double loop-clip to a predefined degree of stenosis, which also reduces trauma on the aorta while improving the reproducibility of the tightness of ligation, and, thus, reducing mortality rates^28,30^. As the current method also employs a needle to standardize vessel diameter with uncontrolled tightening of the ligation, it is likely that these procedures could also be employed to improve rodent abdominal aortic constriction models.

A hallmark of pathological LV hypertrophy is activation of the fetal gene program (*Nppa*, *Nppb*, *Myh7* and *Acta1*) in cardiomyocytes. We observed a more pronounced expression of these genes in enriched cardiomyocytes than in LV tissues (which include non-myocytes) at one week post-SAC. In a separate cohort of mice in which fetal gene expression was evaluated at 24 h after SAC, we observed increased expression of *Myh7* and *Nppb* in the apex of the heart, but not of *Nppa* or *Acta1*, suggesting that *Myh7* and *Nppb* are early markers of PO in the heart. Moreover, pathological LV remodelling was evident by the presence of perivascular, but not interstitial, fibrosis in the LV at one week post-SAC; indicating reactive rather than reparative fibrosis. Interstitial fibrosis is mainly associated with the development of diastolic dysfunction due to stiffening of the LV wall, which was not apparent at one week post-SAC. Perivascular fibrosis occurred under great functional pressure involving major LV wall stress altering the vessel wall mechanics in intramyocardial vessels that is also observed in patients with hypertensive heart disease^31,32^. Although there are limited studies on the coronary anatomy of murine hearts, which is slightly different from that of humans’, it has been shown that C57BL/6J mice have three main coronaries and that their coronary arteries are intramyocardial rather than epicardial as in humans. In these mice, the left, right and septal coronary arteries (the septal artery may originate from either the left or right coronary, or two septal coronaries may coexist from either^33^) supply the left and right ventricles, and interventricular septum, respectively^33–36^. Thus, SAC-induced hypertensive heart disease predominantly involves collagen deposition, due to fibroblast proliferation^32^, around the arteries branching from the left coronary artery into the LV free wall^34,36^ rather than from those located in the interventricular septum (IVS), perhaps indicating greater wall stress due to a higher transmural pressure in the LV free wall than that across the IVS. This involves the compression of intramural LV coronary arteries that may impair coronary flow reserve and lead to ischaemia^37^. Our observation of increased *Hif1a* expression in an enriched preparation of cardiomyocytes, at one week post-SAC, is supportive of a role for ischaemia in the pathophysiology of SAC-induced hypertrophy and fibrosis. Moreover, localization of fibrosis to the perivascular regions of the myocardium might also explain the heterogeneity of *Hif1a* expression observed in single cell RNA-seq studies after one week of TAC-induced PO^38^. Taken together, our findings indicate that SAC causes an increase in LV end-systolic wall stress plus perivascular fibrosis that likely leads to regional differences that are more susceptible to reductions in coronary flow and hypoxia, resulting in heterogeneity in the ischaemic response of cardiomyocytes to PO.

SAC involves banding the abdominal aorta above the origin of both renal arteries that supply the kidney and may result in reduced renal perfusion, which, in turn, may activate the RAS, and therefore, could be considered a RAS-dependent model of LV hypertrophy. In rats, PRA was shown to increase within 15 minutes^39^ post-SAC, while another study showed PRA was elevated at one day but returned to control values after three days^40^. Other studies in rats have observed no increase in PRC after 3 days^41^ or in PRA at 1, 3, 7 or 28 days^42^. Of the limited SAC studies in mice, one reported no increase in PRA or kidney renin mRNA with chronic PO after eight weeks of banding C57BL/6J mice^43^. Interestingly, SAC-induced PO in rats has been reported to enhance angiotensin converting enzyme (ACE) activity at 1–7 days post-SAC, peaking at 3 days, in cardiac tissues, but serum ACE activity levels were unaltered, indicating tissue RAS activation^44^. Similar to these reports, neither PRC nor PRA were enhanced at 24 h post-SAC, indicating that circulating RAS did not contribute to initial pathological LV remodelling at this time. However, we found that renin mRNA was slightly elevated in kidney tissues after one week of SAC relative to shams and as a substrate for RAS may contribute to circulating RAS or local RAS activation in the heart.

From previous observations, inhibition from conception (Kit^W/Wv^ mice) or birth [Tg(αMHC-Kit^*Wv*^)] of c-Kit signalling in cardiomyocytes leads to beneficial outcomes following SAC^18^ or MI^24^, respectively. Here, we show c-Kit mRNA and protein is extremely low in wild-type LV tissues and in enriched cardiomyocyte fractions, and that its expression does not change post-SAC. It is likely that non-myocytes are the source for the low level of c-Kit protein found in homogenates since our cardiomyocyte preparation, although enriched for cardiomyocytes, also contained endothelial cells. Indeed, genetic lineage-tracing models have shown that c-Kit is expressed in a subpopulation of endothelial cells^12^ that double after MI^11^ and that cardiomyocytes rarely express c-Kit or originate from c-Kit^+^ cells^11,13,14^. One lineage-tracing study reported a modest increase in c-Kit-expressing cardiomyocytes following TAC-induced PO, although the increase was below clinically-relevant levels^45^. However, genetic lineage-tracing *Kit-Cre* models that result in haploinsufficiency of the c-Kit gene have been questioned, as reviewed^46–49^. These may be addressed in the future by using a dual recombination system^48^. Our results are in agreement with those of others that expression of c-Kit is barely detectable in adult cardiomyocytes^18,19,50^ and is not changed by cardiomyocyte injury due to isoproterenol administration^50^ or MI^11–13^. In summary, this study found that wild-type murine c-Kit expression in cardiomyocytes is not induced in a SAC model of PO that resulted in increased aortic pressure and pathological hypertrophy, which questions c-Kit signalling as a target for treating myocardial injury in the adult by the reactivation of cardiomyocyte proliferation.

## Methods

### Animals

C57BL/6J wild-type or heterozygous transgenic mice, Tg(αMHC-Kit^*Wv*^), Tg(αMHC-Kit1) or Tg(αMHC-Kit2), were maintained under a 12-h light/dark cycle and given food and water ad libitum. In transgenic mice generated on a C57BL/6J background, wild-type c-Kit, Tg(αMHC-Kit1) or Tg(αMHC-Kit2), or, the dominant-negative c-Kit^Wv^ (T660M) mutant, Tg(αMHC-Kit^*Wv*^)^18,24^, were expressed specifically in cardiomyocytes (under the control of the α-MHC promoter). All studies were approved by the Garvan/St Vincent’s Hospital/Animal Ethics Committee and performed in accordance with the guidelines of the Australian Code of Practice for the Care and Use of Animals for Scientific Purposes.

### Suprarenal aortic constriction surgery

Mice (8–10-week-old males) were anesthetized with 2–4% isoflurane and the abdomen shaved. Body temperature was maintained at 37 °C by securing the animal on a heating pad. After sterilizing the skin with ethanol, a left lateral abdominal incision was made and the muscular abdominal wall retracted for access and visualization of the abdominal aorta, which was gently isolated from surrounding tissues. A curved suture needle with 7.0 silk was inserted under the aorta immediately proximal to the origin of the renal arteries and the aorta ligated by tying the silk tightly down onto a blunt, bent 29 G needle, which was then removed (Supplementary Fig. S1). The diameter of the constricted aorta was approximately one-third of its original size. Sham operations were performed similarly with the aorta being isolated but not ligated. The abdominal muscles were sutured together with 7.0 silk and the skin incision was closed with surgical wound clips and then cleaned with iodine. After surgery, mice were placed in recovery cages on a heating pad, monitored for 24 h and then housed in individual cages. Buprenorphine (0.075 mg/kg, sc) was administered for analgesia, twice daily for three days after surgery. Mice were weighed and monitored each day over the duration of the study. The mortality rate in SAC-operated mice was 27% (3% died during surgery, 12% died within 24 h of surgery, 10% were culled because of ≥ 10% loss in body weight, and 2% culled due to severe systemic oedema consistent with congestive heart failure).

### Echocardiography

As previously described^24^, mice were anesthetized (3–5% isoflurane for induction, 1–2% isoflurane for maintenance) six days after surgery and placed on a heating pad. After shaving the chest and washing with 70% ethanol, pre-warmed ultrasound gel was applied to the chest wall, and echocardiography was performed using a MS400 18–38 MHz transducer probe and VEVO 2,100 ultrasound system (VisualSonics Inc., Canada). Recording were taken in B-mode of the LV long axis (LAX) followed by short axis (SAX) views at the mid papillary level. LV length (L) was measured from the apical dimple to the base of the closed aortic valve leaflets from the LAX images at end-diastole and end-systole. The areas boarded by the endo- and epicardium were determined by planimetry of the SAX image at end-diastole and end-systole, and from this a modified Bullet formula was applied to derive LV end-diastolic and end-systolic volumes (LVEDV and LVESV, where LV vol = L × (endocardial area) × 5/6). Planimetry derived measurements include the area of the papillary muscles. Mean chamber radius (r = √(endocardial area/π) and wall thickness (h = √(epicardial area/π)—r) were calculated at end-diastole. The acquisition of images and evaluation of data were performed by an operator blinded to treatment.

### Micromanometry

Hemodynamic parameters were measured in male C57BL/6J mice (9-week-old) by micromanometry in a blinded manner at one week post-surgery. Mice were anesthetized (3–5% isoflurane for induction, < 2% isoflurane for maintenance) and secured onto a heating pad in the supine position. After shaving the anterior neck and thoracic areas, a midline incision was made on the neck and the right carotid artery isolated with fine-tipped haemostats and forceps. A 1.2 F pressure-sensor catheter (Scisense, Canada) was inserted and the catheter tip advanced into the aortic root for blood pressure recordings and then into the LV to record LV pressure. Pressure traces were acquired and analyzed using AcqKnowledge software (v3.8, Biopac Systems, USA, www.biopac.com/product/acqknowledge-software/). Mice were euthanized by cervical dislocation immediately after micromanometry recordings for tissue collection or heparinized in preparation for cardiomyocyte isolation.

### Tissue collection

Multiple tissues collected from sham and SAC operated mice (9-week-old), including lungs, heart (atria, right and left ventricles), liver and a portion of right kidney were weighed, rinsed in saline, and gently blotted dry before being snap-frozen in liquid nitrogen and stored at − 80 °C. To isolate the left ventricle, atria were removed from the ventricles; the right ventricular wall was accessed by guiding the scissors through the right atrio-ventricular chamber opening to dissect away the right ventricular wall at the boundaries connecting it to the interventricular septal wall, thus leaving the left ventricle intact. Prior to freezing, the left ventricle was dissected into three sections, with the base and apex being processed for protein and RNA experiments, respectively, and the middle section reserved for histology studies (vide infra). The right tibia was collected and stored in 100% ethanol at RT. Tibia length was determined using fine-calibre callipers.

At 24 h post-surgery, hearts (minus the atria) from adult male C57BL/6J mice at 8–10 weeks of age were harvested and divided into two sections, base and apex, and blood collected to prepare plasma (as described below).

### Cardiomyocyte isolation

After micromanometry recordings, cardiomyocytes were collected from sham and SAC hearts by Langendorff retrograde perfusion with Worthington collagenase II^51^, and cardiomyocytes were enriched by multiple differential centrifugations. Each sample (3 ml) was divided into three aliquots: one (2 ml) for Western blotting, one (0.8 ml) for RT-qPCR, both snap-frozen in liquid nitrogen and stored at − 80 °C for downstream extraction of protein or RNA, respectively, and one (0.2 ml) for immunocytochemistry, fixed in 2% PFA for 5 min and subsequently stored in PBS for staining.

### Immunocytochemistry

Fixed cells were prepared by cytospin onto slides (500 rpm, 5 min), incubated first with blocking buffer (1% BSA and 0.2% Triton X-100 in PBS; 1 h, RT), then rabbit anti-laminin antibody (cat. #: ab11575, Abcam, diluted 1:200 in blocking buffer; overnight, 4 °C) and washed (× 5 in PBS, 5 min, RT). The following steps were performed in the dark: slides were incubated with goat anti-rabbit IgG Alex Fluor 555 (diluted 1:1,000 in blocking buffer; 1 h, RT) and washed (× 5 in PBS, 5 min, RT) before staining of nuclei with DAPI (cat #:D9542, Sigma, diluted 1:5,000; 10 min, RT) and washing (× 5 in PBS, 5 min, RT). Coverslips were secured with PVA-DABCO mounting medium and images were acquired on a Zeiss AxioImager Z1 fitted with a LSM700 confocal scan head.

### Cardiomyocyte area

The area of fixed binucleated cardiomyocytes was determined using ImageJ by transforming immunocytochemistry data into a binary image, which allowed automated outlining of cells from which the planimetry area was calculated relative to the scale bar. Overlapping cardiomyocytes were excluded from analyses that was performed in a blinded manner.

### RT-qPCR

RT-qPCR was performed by TaqMan (TaqMan Gene Expression Master Mix, Thermo Fisher) or SYBR Green assay (LightCycler 480 SYBR Green I Master, Roche) following cDNA synthesis (Superscript III First Strand Synthesis System or QuantiTect Reverse Transcription Kit, respectively). The following TaqMan probes (Thermo Fisher) were used: *Nppa* (Mm01255747_g1), *Nppb* (Mm01255770_g1), *Myh7* (Mm00600555_m1), *Acta1* (Mm00808218_g1), *Hprt* (Mm01545399_m1), *Hif1a* (Mm00468869_m1), *Vegfa* (Mm01281449_m1), *Kit* (Mm00445212_m1). For SYBR Green assays *Renin* (forward: 5′-GAG GCC TTC CTT GAC CCA TC-3′, and reverse: 5′-TGT GAA TCC CAC AAG CAA GG-3′) and *Gapdh* (forward: 5′-CTT GGG CTA CAC TGA GGA C-3′, and reverse: 5′-CTG TTG CTG TAG CCG TAT TC-3′) primers were used from Integrated DNA Technologies.

### Histology

The middle section of LVs were immersed in fresh 2% PFA (4 h, RT), stored in 70% ethanol (RT) and embedded in paraffin. Tissues were sectioned (5 μm, Leica RM2255 microtome), deparaffinized and rehydrated. Nuclei were stained with Weigert’s iron haematoxylin (8 min, RT; cat #: HT1079, Sigma) in a dark humidity chamber, washed in running deionized water (10 min) and stained with 0.1% Sirius red F2B (cat #: 365548, Sigma) and 0.1% Fast Green FCF (cat #: F7258, Sigma) in saturated picric acid (1 h, RT; cat #: P6744, Sigma) in a dark humidity chamber to identify collagen fibres (type I and III) and cytoplasm, respectively. Sections were rinsed in 0.5% v/v glacial acetic acid followed by deionized water, then dehydrated and mounted in DPX. Images were taken with Leica DM6000B light microscope (power mosaic at 20 × objective).

### Plasma renin activity and concentration measurements

Blood was collected by cardiac puncture at 24 h after sham or SAC surgery and mixed by inversion with Na_2_EDTA (1–2 mg/ml) in 1.5 ml microfuge tubes followed by centrifugation at 12,000 *g* for 10 min at 4 °C. The plasma supernatant was collected into aliquots and stored at − 80 °C. Plasma renin concentration (PRC) and activity (PRA) were analyzed in duplicate using a radioimmunoassay service (ProSearch International, Australia) and users were blinded to the sample identity. PRC was determined by the generation of Ang I in the presence of the exogenous sheep renin substrate, angiotensinogen. PRA was measured using endogenous levels of renin substrate and any Ang I that was already present in the plasma was subtracted^52^.

### Immunoprecipitation and immunoblotting

Cardiomyocytes and heart tissues were lysed in cold lysis buffer (50 mM Tris–Cl, pH 7.5; 150 mM NaCl; 1 mM Na4P2O7; 1 mM Benzamidine; 5 mM Na_3_VO_4_; 10 mM NaF; including protease inhibitors, #11836170001, Roche, 4 °C) using a polytron homogenizer. Lysates were incubated (45 min, 4 °C, gentle agitation) and centrifuged (12,000 rpm, 15 min, 4 °C). Protein concentrations of supernatants were determined (Direct Detect, Merck Millipore). Lysates were pre-cleared (protein A and G magnetic beads; cat. # 16–663, Millipore) and incubated with antibody-conjugated beads (overnight, 4 °C). Bound fractions were eluted by magnetic separation (MagnaRack) and resuspended in lysis buffer containing 0.5% NP-40 (20 µl). Following immunoprecipitation of c-Kit (anti-c-kit M14 antibody, 1:50 dilution; Santa Cruz), lysates and immunoprecipitates were subjected to SDS-PAGE (XCell SureLock Mini-Cell Electrophoresis System, Thermo Fisher) and blotted onto PVDF membranes. Elution blots were incubated with anti-c-Kit D13A2 antibody (1:500 dilution; Cell Signaling) and lysate blots were incubated with anti-GAPDH antibody (1:2000; cat #: 2,118, Cell Signaling; 1 h, RT). Blots were then incubated with species-specific antibodies conjugated to horseradish peroxidase (HRP, 1:4,000–1:10,000 dilution, 1 h, RT), developed with chemiluminescent substrates (low HRP sensitivity Western Lightning ECL, Perkin Elmer; or, high HRP sensitivity Pierce ECL Plus, Thermo Fisher, Supplementary Fig. S3), exposed to X-ray film (Hyperfilm ECL; Amersham) and films were developed (SRX-101A processor; Konica Minolta). Densitometry (ImageJ) was used to normalize c-Kit protein pixel number to GAPDH pixel number.

### Data processing and statistical analysis

Data are expressed as means ± standard deviations. Sham and SAC data were passed through Kolmogorov–Smirnov test for normality, and a two-tailed Student’s unpaired t-test was performed on normally distributed data (Welch’s correction was applied if standard deviations were unequal). If data were nonparametric then a Mann–Whitney t-test was performed. P < 0.05 was considered statistically significant. Statistics were performed using GraphPad Prism version 8.3.1 for Windows (GraphPad Software, San Diego, California USA, www.graphpad.com).

LV Triple product (LVTP)^53^, end-systolic pressure (ESP)^54^, LV ejection fraction (EF %), fractional shortening (FS %), LV end-systolic wall stress (LVESWS)^55^, and LV end-diastolic wall stress (LVEDWS)^55^ were calculated using the following equations:

- LVTP = LVHR x LVSP x dP/dt_max_,
- ESP = (SAP × 2 + DAP) / 3,
- LVEF = (LVEDV – LVESV) / LVEDV,
- FS = (LVEDD – LVESD) / LVEDD,
- LVESWS = ESP x R_end-systole_ / (2 × h_end-systole_),
- LVEDWS = LVEDP x R_end-diastole_ / (2 × h_end-diastole_).

*HR* heart rate, *SAP* peak systolic aortic pressure, *DAP* end-diastolic aortic pressure, *SV* stroke volume, *LVEDV* LV end-diastolic volume, *LVEDD* LV end-diastolic dimension, *LVESD* LV end-systolic dimension, *R* LV internal chamber radius, *h* LV wall thickness.

## Supplementary information

Supplementary file1
