# Supplementary material for: Pressure overload by suprarenal aortic constriction in mice leads to left ventricular hypertrophy without c-Kit expression in cardiomyocytes
Source: Sci Rep. 2020 Sep 18;10:15318. doi: 10.1038/s41598-020-72273-3 (PMC7501855; doi:10.1038/s41598-020-72273-3)
Supplement: Supplementary file 1 — Supplementary file1 [file 41598_2020_72273_MOESM1_ESM.pdf]

Pressure overload by suprarenal aortic constriction in mice leads to left ventricular hypertrophy without c-Kit expression in cardiomyocytes

Amy M Nicks, Scott H Kesteven, Ming Li, Jianxin Wu, Andrea Y Chan, Nawazish Naqvi, Ahsan Husain, Michael P Feneley, Nicola J Smith, Siiri E Iismaa & Robert M Graham*

Supplementary information

***Corresponding author:**

Robert M Graham, Division of Molecular Cardiology and Biophysics, Victor Chang Cardiac Research Institute, 405 Liverpool Street, Darlinghurst, NSW 2010, Sydney, Australia, Tel: +61 (0)2 9295 8677, E-mail: b.graham@victorchang.edu.au

Supplementary methods

Immunocytochemistry for supplementary data

Fixed cells were prepared by cytospin onto slides (500 rpm, 5 min), incubated first with blocking buffer (5% goat serum and 0.1% Triton X-100 in PBS; 1 hr, RT), then mouse anti-myosin heavy chain (cat. #: ab 50967, Abcam, diluted 1:800) and rabbit anti-c-Kit D13A2 (cat. #: 3074S, Cell Signaling, diluted at 1:600) overnight at 4°C. Samples were washed (x5 in PBS, 5 min, RT). The following steps were performed in the dark: slides were incubated with species-specific secondary antibodies, goat anti-mouse IgG Alexa Fluor 488 and goat anti-rabbit Alexa Fluor 594 (diluted at 1:1000) for 1 hr at RT, and washed (x5 in PBS, 5 mins, RT). Coverslips were secured onto each slide with hard mounting medium containing DAPI and images were acquired on a Zeiss AxioImager Z1 fitted with a LSM700 confocal scan head.

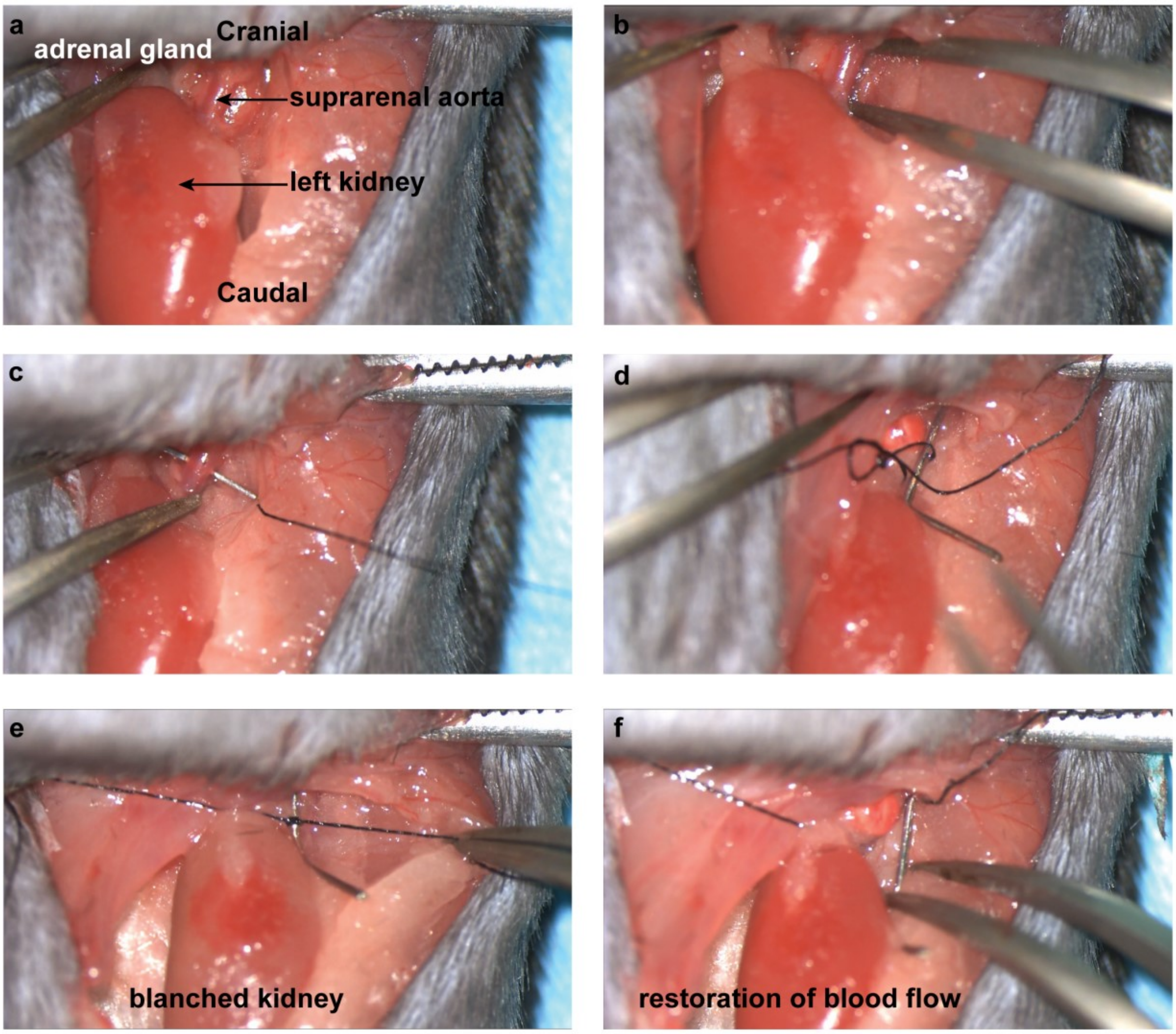

Supplementary Figure S1: SAC surgery

Representative images taken during SAC surgery showing (**a**) the left kidney and the aorta exposed at the suprarenal level after a lateral incision; **b**, the aorta isolated from surrounding tissue using forceps; **c**, a suture needle used to guide the 7.0 silk around the aorta; **d**, silk looped twice around the aorta and a blunted 29 G needle; **e**, blood flow was occluded blanching the kidney after a double knot was tightened; **f**, partial blood flow was restored after removal of the needle.

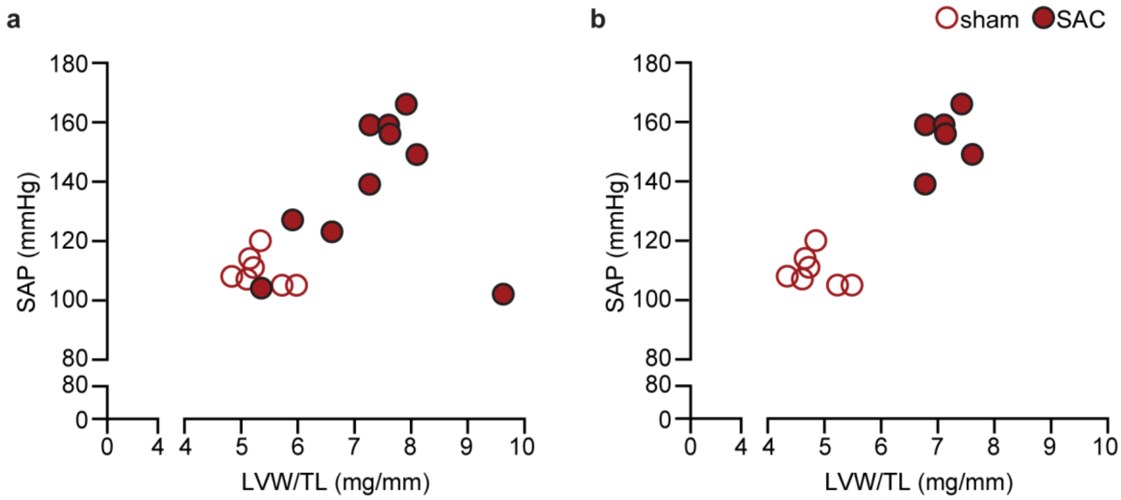

Supplementary Figure S2: SAP and LVW/TL before and after exclusion criteria are applied

Pearson's correlation between SAP and LVW/TL ratio in sham- or SAC- operated mice one week after surgery. **a**, shows all sham- and SAC-operated mice (n=17), R^2=0.54; and (**b**) shows SAC-operated animals that met the exclusion criteria of SAP <129 mmHg (>2 standard deviations above the sham mean SAP of 109 ± 10 mmHg), (n=13) R^2=0.92. Sham data are the same in **a** and **b** (n=7). Three of the four SAC-operated mice excluded from this analysis had comparable SAPs and LV weights to sham controls. The fourth normotensive SAC mouse (SAP=105 mmHg) excluded from analysis had a marked increase in LV weight (LVW/TL, +90%) and LV wall thickness, but diminished cardiac contractility (dP/dT max and min) compared to shams, indicating that this animal had rapidly advanced into heart failure (also observed in a TAC model[1]). SAP, peak systolic aortic pressure; LV, left ventricle; TL, tibia length.

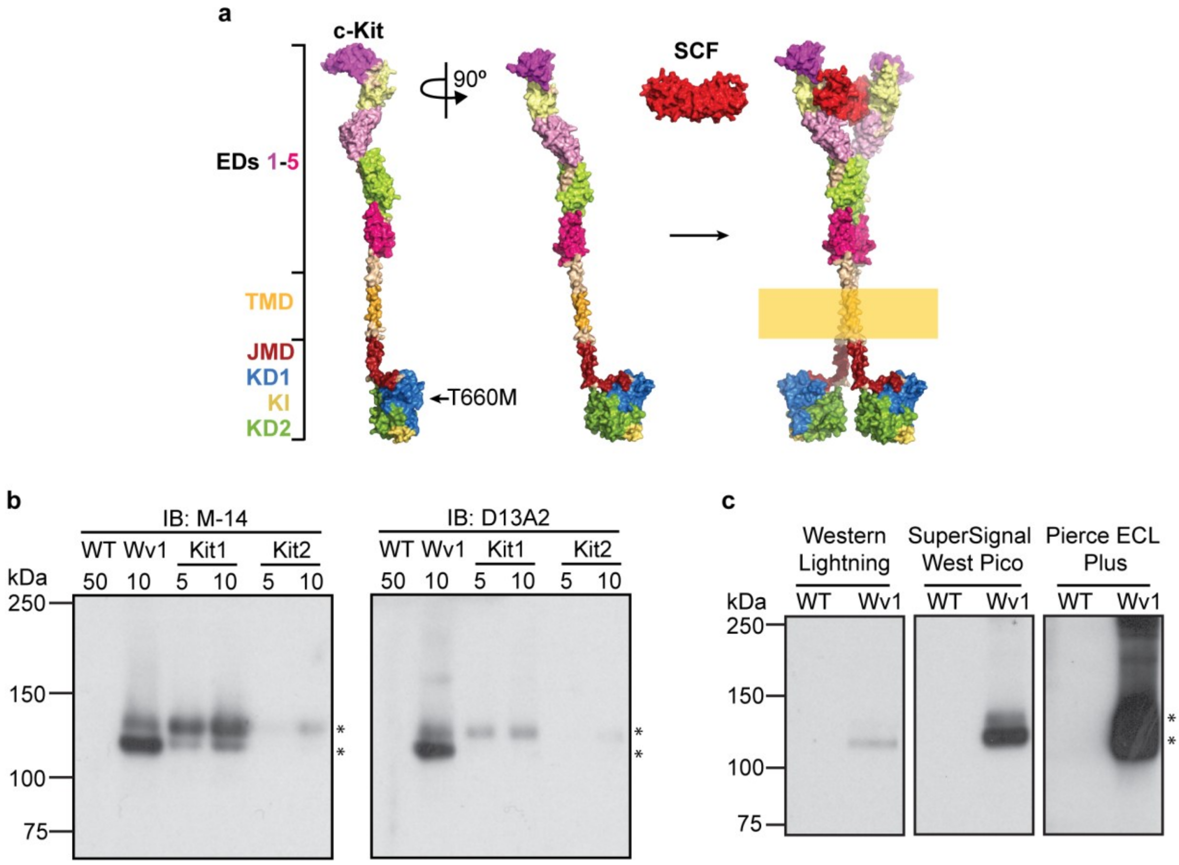

Supplementary Figure S3: c-Kit protein structure and detection

a, c-Kit has five extracellular Ig-like domains (EDs), a transmembrane (TMD), a juxtamembrane domain (JMD), two kinase domains (KD) 1 and 2 (a N-lobe and C-lobe) divided by a kinase insert (KI), and a C-terminal domain. Soluble dimeric stem cell factor (SCF) binds to two c-Kit monomers, resulting in c-Kit dimerization and autophosphorylation of intracellular tyrosines activating downstream signaling pathways. Tg(αMHC-Kit^{Wv}) mice have a missense mutation at T660M located in kinase domain 1, but the effect on the protein structure from the c-Kit mutation is unresolved. These models were made using Protein Data Bank IDs: 2e9w[2], 2ec8[2], 1pkg[3], and generated using PyMol. **b**, Wild-type, WT, 50 µg; Tg(αMHC-Kit^{Wv}), Wv1, 10 µg; Tg(αMHC-*Kit.1*), Kit1, 5 and 10 µg); and Tg(αMHC-*Kit.2*), Kit2, 5 and 10 µg heart lysates were separated by SDS-PAGE and c-Kit detected using two different anti-c-Kit antibodies, M-14 (1:1000) or D13A2 (1:500), followed by secondary horseradish peroxidase (HRP) antibodies (1:4000) and detection using Western Lightning ECL. Films were exposed to membranes probed with M-14 or D13A2 for 10 seconds or 3 minutes, respectively. *indicates c-Kit species at approx.125 and 145 kDa. The predominance of the smaller immature species of c-Kit is evident in Tg(αMHC-Kit^{Wv}) heart lysates compared to the predominance of the larger mature species of c-Kit in Tg(αMHC-*Kit.1*) and

Tg(αMHC-*Kit.2*) heart lysates. **c**, WT and Tg(αMHC-Kit^{Wv}) heart lysates (100 and 20 μg, respectively) were separated by SDS-PAGE, followed by Western blotting and c-Kit protein was incubated with anti-c-Kit M14 antibody (1:1000), followed by the same secondary HRP antibody that was diluted as per manufacturer's recommendations for each ECL kit: 1:5000 for Western Lightning ECL (Perkin Elmer), and at 1:25000 for SuperSignal West Pico (Thermo Fisher) and Pierce ECL Plus (Thermo Fisher). Films were exposed to blots for 1 second. The order from highest to lowest HRP sensitivity is Pierce ECL Plus > SuperSignal West Pico > Western Lightning ECL.

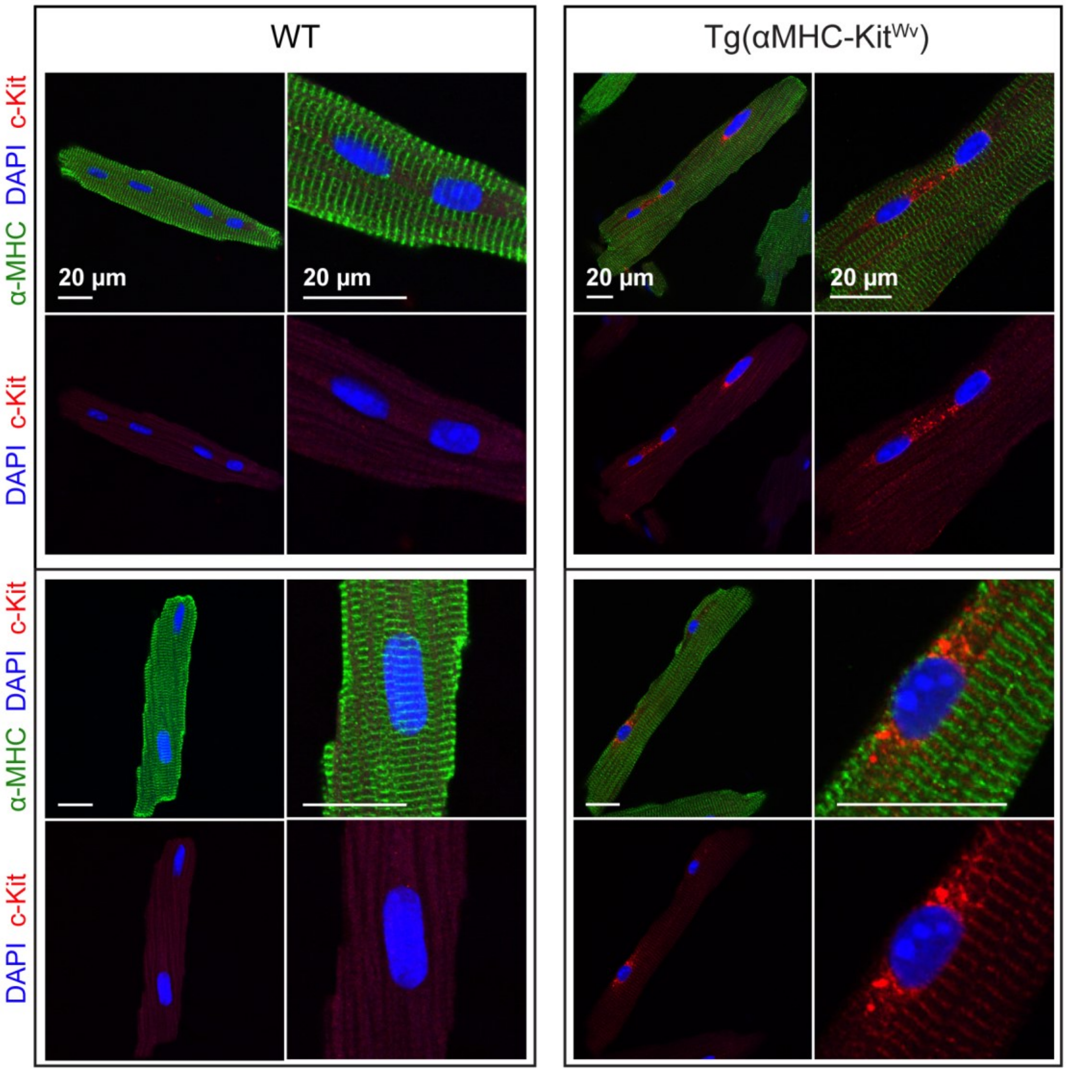

Supplementary Figure S4: c-Kit expression in Tg(αMHC-Kit^{Wv}) cardiomyocytes

Representative C57BL/6J wild-type, WT, and Tg(αMHC-Kit^{Wv}) adult cardiomyocytes were co-stained with the cardiomyocyte-specific marker, α- myosin heavy chain (α-MHC, green), and for c-Kit with the anti-c-Kit antibody D13A2 (red), and nuclear DNA with DAPI (blue), images were acquired by confocal microscopy, as described in the Methods. c-Kit appears in the perinuclear region of Tg(αMHC-Kit^{Wv}) cardiomyocytes. Scale bar is 20 μm Together, the predominance of the smaller immature species of c-Kit in Tg(αMHC-Kit^{Wv}) cardiomyocytes determined by Western blot (Supplementary Fig. S3) suggests the c-Kit^{Wv} protein may be misfolded and trapped in the endoplasmic reticulum.

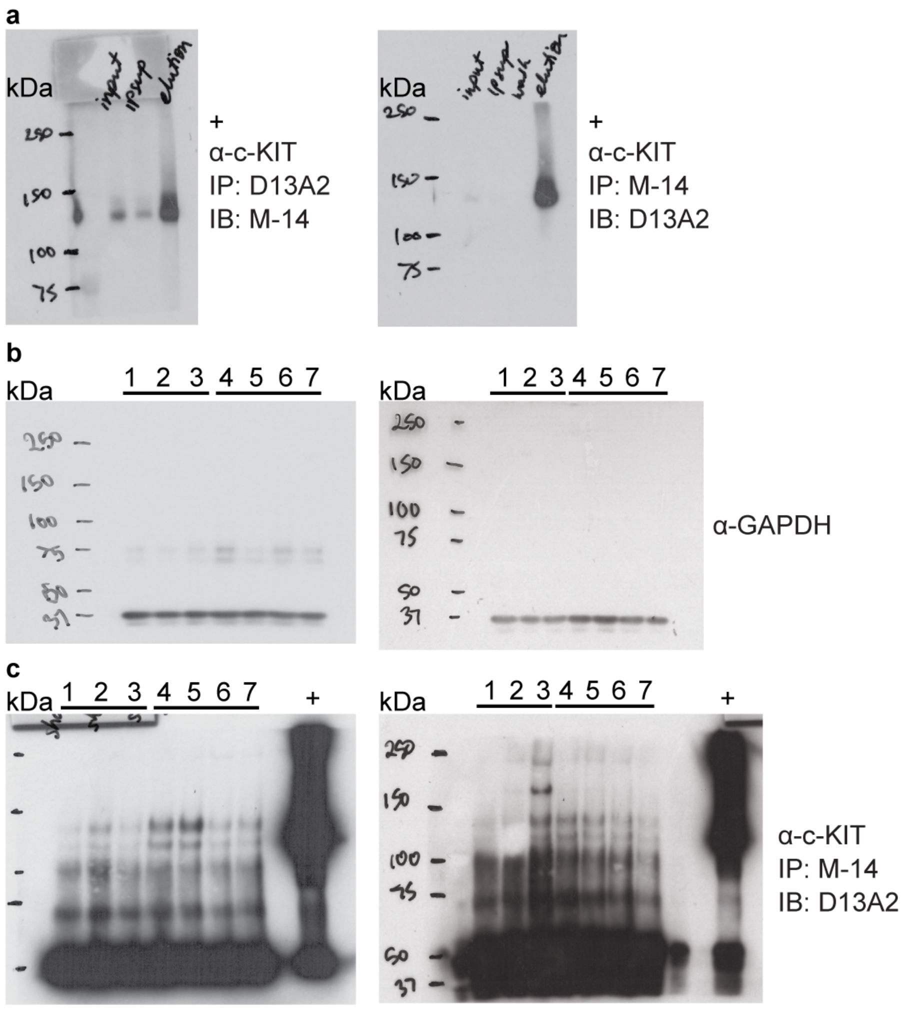

Supplementary Figure S5: Full-length Western blots

a, Anti-c-Kit antibody (Ab) specificity was demonstrated by immunoprecipitation (IP) of heart lysates (400 µg) from transgenic mice overexpressing the dominant negative *Wv* c-Kit mutant, Tg(αMHC-Kit^{Wv}; positive control "+"), using either anti-c-Kit D13A2 or M-14 Ab (1:50) followed by size-fractionation and immunoblotting (IB) with the other anti-c-Kit Ab M-14 (1:1000) or D13A2 (1:500), followed by secondary horseradish peroxidase (HRP) antibodies (1:4000) and detection using Western Lightning ECL (low HRP sensitivity, Supplementary Fig. S3). Films were exposed to blots for 1 minute. Inp., IP input (20 µl); IP sn., IP supernatant (20 µl); IP wash (20 µl); Elute, IP elution from beads (20 µl). **b**, c-Kit and GAPDH were quantified by densitometry. **c**, c-Kit protein proteins were detected using Pierce ECL Plus (high HRP sensitivity, Fig. S3) at one-week post–sham (wells 1-3; n=6) or –SAC surgery (wells 4-6; well 7 is a SAC cardiomyocyte lysate but blood pressure was normotensive; n=6) from C57BL/6J adult male mice (9-week old). Films were exposed to blots for 10 minutes.

References

1. Furihata, T. *et al.* The experimental model of transition from compensated cardiac hypertrophy to failure created by transverse aortic constriction in mice. *IJC Hear. Vasc.* **11**, 24–28 (2016).

2. Yuzawa, S. *et al.* Structural basis for activation of the receptor tyrosine kinase KIT by stem cell factor. *Cell* **130**, 323–334 (2007).

3. Mol, C. D. *et al.* Structure of a c-Kit product complex reveals the basis for kinase transactivation. *J. Biol. Chem.* **278**, 31461–31464 (2003).
